# Advances in Preclinical/Clinical Glioblastoma Treatment: Can Nanoparticles Be of Help?

**DOI:** 10.3390/cancers14194960

**Published:** 2022-10-10

**Authors:** Daniel Ruiz-Molina, Xiaoman Mao, Paula Alfonso-Triguero, Julia Lorenzo, Jordi Bruna, Victor J. Yuste, Ana Paula Candiota, Fernando Novio

**Affiliations:** 1 Catalan Institute of Nanoscience and Nanotechnology (ICN2), CSIC and BIST, Campus UAB, Bellaterra, 08193 Barcelona, Spain; 2 Institut de Biotecnologia i de Biomedicina, Departament de Bioquimica i Biologia Molecular, Universitat Autònoma de Barcelona, 08193 Cerdanyola del Vallès, Spain; 3 Departament de Bioquímica i Biologia Molecular, Universitat Autònoma de Barcelona, 08193 Cerdanyola del Vallès, Spain; 4 Neuro-Oncology Unit, Bellvitge University Hospital-ICO (IDIBELL), Avinguda de la Gran Via de l’Hospitalet, 199-203, L’Hospitalet de Llobregat, 08908 Barcelona, Spain; 5 Instituto de Neurociencias. Universitat Autònoma de Barcelona (UAB), Campus UAB, 08193 Cerdanyola del Vallès, Spain; 6 Centro de Investigación Biomédica en Red Sobre Enfermedades Neurodegenerativas (CIBERNED), Campus UAB, 08193 Cerdanyola del Vallès, Spain; 7 Centro de Investigación Biomédica en Red: Bioingeniería, Biomateriales y Nanomedicina (CIBER-BBN), 08193 Cerdanyola del Vallès, Spain; 8 Departament de Química, Universitat Autònoma de Barcelona (UAB), Campus UAB, 08193 Cerdanyola del Vallès, Spain

**Keywords:** glioblastoma, preclinical model, nanoparticles, drug delivery, brain cancer, BBB

## Abstract

**Simple Summary:**

As one of the most lethal human cancers, glioblastoma treatment is a real challenge because of several resistance mechanisms, including limited drug entry into the central nervous system through the blood–brain barrier and the vast heterogeneity of this family of tumors. In the development of precision medicine, various nanoconstructs are being proposed to cross the BBB, specifically target GB tumors, release the therapeutic cargo in a controlled manner, and reduce therapeutic resistance. This review summarizes the different families of nanoparticles and approaches followed so far pursuing these aims.

**Abstract:**

Glioblastoma multiforme (GB) is the most aggressive and frequent primary malignant tumor in the central nervous system (CNS), with unsatisfactory and challenging treatment nowadays. Current standard of care includes surgical resection followed by chemotherapy and radiotherapy. However, these treatments do not much improve the overall survival of GB patients, which is still below two years (the 5-year survival rate is below 7%). Despite various approaches having been followed to increase the release of anticancer drugs into the brain, few of them demonstrated a significant success, as the blood brain barrier (BBB) still restricts its uptake, thus limiting the therapeutic options. Therefore, enormous efforts are being devoted to the development of novel nanomedicines with the ability to cross the BBB and specifically target the cancer cells. In this context, the use of nanoparticles represents a promising non-invasive route, allowing to evade BBB and reducing systemic concentration of drugs and, hence, side effects. In this review, we revise with a critical view the different families of nanoparticles and approaches followed so far with this aim.

## 1. Glioblastoma Treatment: State-of-the-Art

**Figure 1:**
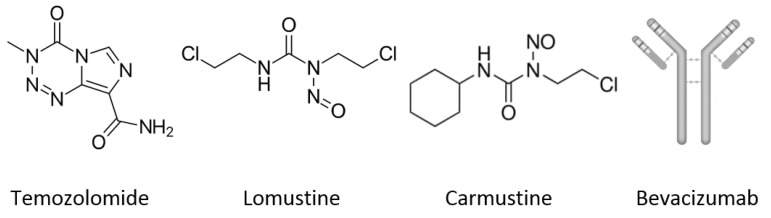
First-line (TMZ) and second-line (lomus, carmustine and BVZ) drugs for glioblastoma treatment.

Glioblastoma (GB) is a devastating tumor representing more than 50% of primary malignant brain tumors [1], and has been classified as having the highest malignant grade (4) behavior by the World Health Organization (WHO) [2,3]. Its expected median overall survival is around 15 months even after aggressive therapy. The standard treatment entails surgical resection and radiotherapy, combined with oral temozolomide (TMZ) chemotherapy [4,5], as validated in a randomized clinical trial in 2005 [6]. Its therapeutic mechanism involves methylation of the guanine O6 position and subsequent DNA double-strand rupture, leading to a cell cycle arrest and cell death [7,8]. Additional benefits of TMZ can be immunogenic cell damage and exposure of calreticulin immunogenic signals [9]. On the negative side, TMZ must be administered at high doses and prolonged periods due to its low yield crossing the blood–brain barrier (BBB), short half-life, inducing side effects [10,11], and an intrinsic resistance to DNA repair [12,13]. Alternatively, second-line treatments involving nitrosourea-based drugs such as lomustine (lomus) or carmustine were approved by the US Food and Drug Administration (FDA) and European Medicines Agency (EMA) [14,15]. These drugs are also effective alkylating agents inducing cross-linking between DNA strands and protein carbamylation that prevent cell cycle development [16]. However, remarkable side effects may appear, such as myelosuppression, hepatic toxicity, and pulmonary fibrosis [17]. In addition, the antiangiogenic drug Bevacizumab (BVZ, a recombinant humanized monoclonal antibody acting through inhibition of the vascular endothelial growth factor A) was also FDA approved for tumor recurrence [18], yet clinical trials combining unlike schedules/combinatory treatments [19] and immune checkpoint inhibitors (ICIs) [20] revealed disappointing efficacy results (Figure 1).

Finally, platinum (Pt) complexes appear as a potential fourth line of GB treatment. These agents are known to form DNA adducts that avoid both cell replication and transcription [21], inducing DNA damage and cell apoptosis with good effectiveness for diverse cancers [22]. However, inconsistent results are obtained in the treatment of GB patients mainly due to systemic toxicities [23] occurring before effective drug concentrations reach the tumor [24,25,26]. Other antiangiogenic agents, such as integrins, transmembrane receptors overexpressed on both GB and tumor-associated endothelial cells [27], or tyrosine kinase inhibitors likewise represent complementary therapeutic approximations. However, none of these therapeutic approaches showed relevant advantages after being tested in phase 2–3 trials [28,29,30,31,32].

Challenges of crossing natural barriers: BBB represents a vasculature for preserving brain homeostasis and neurological functions, being permeable to only 2–3% of small molecules (large molecules are excluded). Additionally, it has functional efflux pumps for clearance of toxic, metabolic, and other waste products. The BBB consists of a neurovascular unit (NVU) that includes a basement membrane, pericytes, astrocytes, and capillary endothelial cells connected to each other by tight junctions. However, when cancer cells displace endothelia from the other NVU cells, the BBB breaks down and both passive and active transport are altered, giving rise to the blood–tumor barrier (BTB). Although the BTB is more permeable than the BBB, its permeability to molecules including drugs is heterogeneous, representing a great challenge for therapy. The BTB is accompanied by a decrease in the expression of tight junctions, and increased secretion of vascular endothelial growth factor (VEGF) from tumor cells, as well as a breakdown of the basal membrane. Transport occurs to a very limited extent mostly in disrupted BBB. In a similar way, passive diffusion is uncommon and only for small lipophilic compounds. Worth of mention, endogenous molecules pass through specific transporters. Cell-mediated and adsorption-mediated transport occur as well, but receptor-mediated transport is the most efficient mechanism for drug delivery, being one of the strategies used in nanomedicine approaches. Some of the receptors found on the luminal side of the BBB are transferrin receptor (TfR), insulin and insulin-like growth factor receptor, low-density lipoprotein receptor (LDLR), low-density lipoprotein receptor-related protein 1 and 2 (LRP1 and LRP2), scavenger receptor class B type I (SR-B1), leptin receptor, and lactoferrin receptor. More recently, nicotinic acetylcholine receptors (nAChRS) and diphtheria toxin receptor have been also described [33].

**Figure 2:**
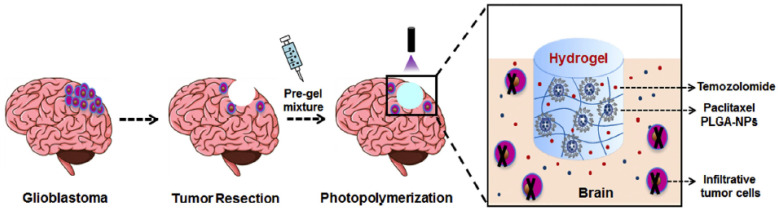
Codelivery of PTX and TMZ through a photopolymerizable hydrogel for the postresection treatment of glioblastoma. Reproduced from Ref. [46] with permission of the copyright holder.

All in all, systemic toxic effects may arise before effective drug concentrations are reached within the tumor, and this fact limits chemotherapy efficacy in GB treatment. This is mostly, though not exclusively, due to difficulties in crossing the BBB, reason why different approaches have been designed to overcome this barrier while minimizing side effects [34]. These strategies include methods to increase the BBB permeability [35,36,37], use of biodegradable polymeric implants in the tumor bed [38], or more aggressive approaches, such as the use of a catheter to deliver drugs directly into the brain through convection-enhanced delivery (CED) [39,40,41]. Gliadel^®^ wafer was also approved by the US FDA back in 2003 as a local implant depot of active ingredient carmustine (BCNU) to treat malignant and recurrent high-grade gliomas (HGG). Soft materials, such as films, fibers, and (hydro)gels, have also been investigated as local implanting depots for different therapeutic agents mostly in preclinical studies [42,43,44,45]. An interesting example is that of Zhao et al., who reported the codelivery of paclitaxel (PTX) and TMZ through a photopolymerizable hydrogel able to synergistically inhibit tumor growth and prevent GB recurrence after surgical resection (Figure 2). The drug combination showed high tolerability and the suppression of tumor growth more efficiently than the administration of single drugs in a U87MG orthotopic tumor model [46]. However, in spite of these pioneering and successful results, formulations that need local device placements for drug delivery are not very attractive from a clinical point of view. These approaches require a new neurosurgical intervention at the relapsing tumor scenario, in patients with in general poor life expectancy and usually handicapped neurologic status. These devices can also hamper neuroimaging, a key point in the assessment of treatment response. In addition, such devices may trigger new local serious adverse events, which the physicians are not familiarized with, as spotlighted by the use of carmustine wafers. These inconveniences preclude these patients to be candidates for other clinical trials, limiting its attractiveness with disappointing clinical performances [47,48]. All in all, the development of innovative approaches that ensures proper and efficient chemotherapeutic treatments still represents an urgent challenge nowadays.

## 2. Nanoparticles for Glioblastoma

**Figure 3:**
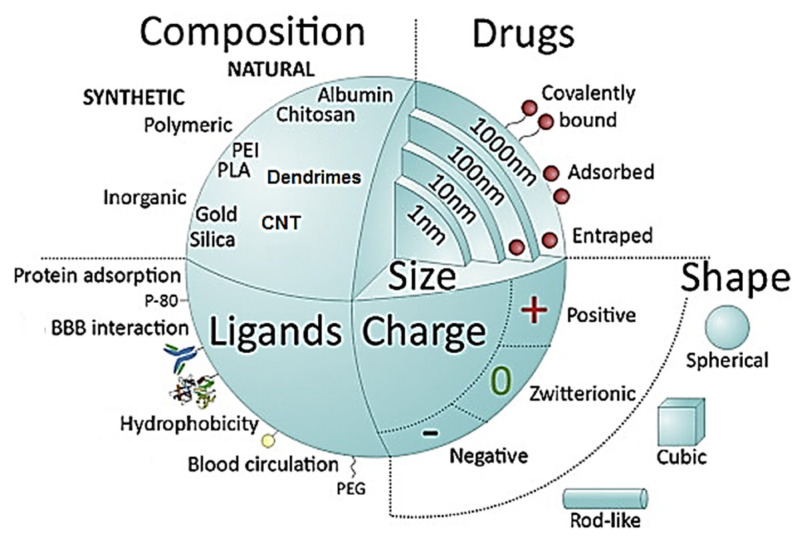
Key nanoparticle (NP) characteristics conditioning drug release and BBB crossing, classified in function of their composition (synthetic or natural), capacity for drug uploading, size, shape, charge, and possibility of surface functionalization to increase their biocompatibility and therapeutic efficacy. Reproduced from Ref. [53] with permission of the copyright holder.

One of the most promising approaches to increase the intravenous (i.v.) local delivery of drugs into the brain has been the use of nanoparticles (NPs) [49,50,51,52,53]. A schematic representation of the NP characteristics that define their efficiency for drug release is shown in Figure 3. Due to their small size and large surface area, nanoparticles can increase the solubility and bioavailability.

The other main advantages of their use are:

- They can endorse BBB diffusion [54] and increase the delivery of higher chemotherapeutic doses (typically restricted by systemic toxicity) to the brain parenchyma [55,56], facilitated in some specific cases with the use of targeting receptors [57];
- They can incorporate additional fluorescent/MRI/radioactive compounds that allow the non-invasive monitoring of its biodistribution [58];
- They confer chemical protection to the drug and a theoretical control over the release upon activation with a stimulus that minimize undesired side effects [59];
- They increase solubility of hydrophobic drugs while favoring a proper biodistribution [60], evading the mononuclear phagocyte system (MPS) [61,62,63,64];
- They can combine different additional therapeutic approaches, such as, not exclusively, radiotherapy sensitization, immune cells stimulation, or induction of heat/radical oxygen species (ROS) [65];
- They can benefit from the well-known enhanced permeability and retention effect (EPR) to access (and remain on) tumor tissues;
- Nanocapsulation increases the half-life activity, for instance in the case of TMZ-loaded chitosan NPs from 1.8 to 13.4 h [66].

**Figure 4:**
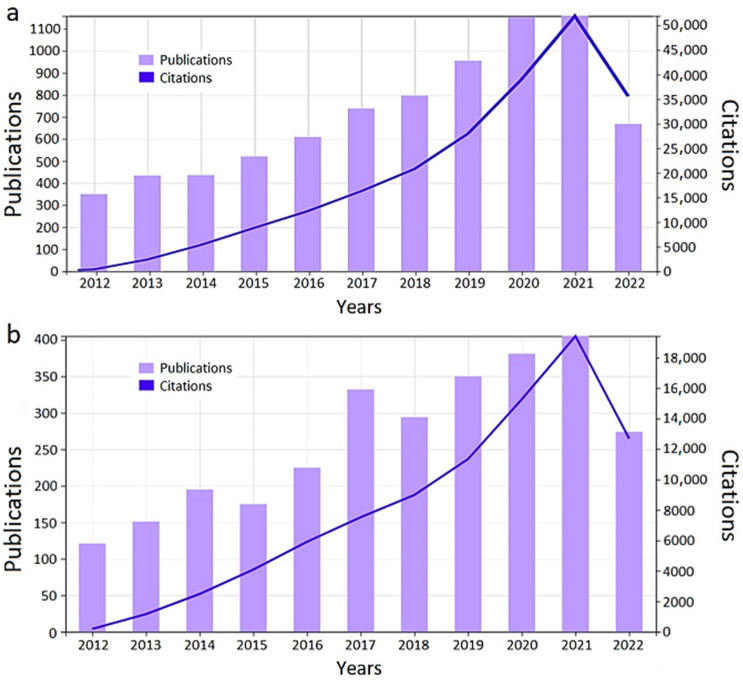
Number of publications and citations in the last 10 years related to (**a**) nanoparticles used for brain diseases and (**b**) nanoparticles developed for glioblastoma treatment.

All these advantages have raised an increasing interest for the development of nanoparticles for brain diseases, as reflected by the increasing number of publications and citations in the area for the last 10 years (Figure 4a). Specifically, for glioblastoma diagnosis and/or treatment (Figure 4b), the 195 articles published in 2010 contrast with more than 400 published in 2021, and allow predicting an upward trend for next years. The data shown in Figure 4b reflects the number of articles mentioning “nanoparticles + glioblastoma” and corresponding citations consulted in the WoS webpage until September 15, 2022. The related publications show different families of NPs that have been used for diagnosis and/or treatment of diseases in the central nervous system (CNS) [65,67,68,69,70], mainly polymeric and lipid-based NPs [71,72]. Drug encapsulation can take place by either physical entrapment, covalent linking or surface adsorption combined with passively [73,74,75] or actively [76,77,78,79] design-dependent targeting. Other frequently used carriers are vesicles (lipidic, micellar, or exosomes) [80,81,82,83,84], mesoporous silica [85], metal nanoparticles [86,87], carbon dots [88], nano-implants [89], or dendrimers [90]. Overall, the use of NPs for GB therapy [91] aims to increase the efficacy of drugs both at preclinical and clinical stages [92].

## 3. Preclinical Studies

Preclinical studies enable researchers to model drug biological effects and predict treatment outcomes (efficacy) as well as toxicity and adverse events (safety) in human patients. While different comprehensive studies have already been reported [93,94,95], in this review, we aim to launch a critical comparison of the different examples so far described in the literature; most of them summarized in Table 1.

As it can be seen there, the experimental design is highly variable across different studies, which makes comparisons extremely difficult. On top of that, nanoparticle batch variations and aspects such as endotoxin amounts are relevant, especially in the case of intravenous administrations [96]. Considering such scattering of experimental parameters, particular attention has been paid to the following relevant aspects:

1. Orthotopic GB studies. Glial tumors are characterized by their heterogeneity and their immunosuppressive tumor microenvironment, which can be hardly replicated in a heterotopic model (e.g., subcutaneous). Such “niches”, comprising all components of a tumor as well as its interaction with tumor microenvironment, must be considered as it might play a role in the therapeutic efficacy [97]. Thus, useful translational studies of relevance in subsequent clinical cases should replicate as faithfully as possible the human situation.
2. Animal and gender model. Regarding species, circa two-thirds of the studies were performed in mice, while the remaining ones have used rats as experimental subjects. With respect to gender of the preclinical subjects, it is worth mentioning that it was not detailed in almost one-third of the studies, and only one of the mentioned papers included representation of both sexes. As for the rest, males are slightly more represented than females, but it is still quite balanced. Overall, it was reported that glioblastoma growth and aggressiveness may vary between males and females [98], so a lack of this information in part of the published studies can lead to a biased information [99].
3. Administration schedule. Administration schedule and methodology used is quite different along the studies shown in Table 1. Regarding the therapy starting point, circa one-third of the studies have started therapy ranging 1–6 days post cell inoculation. The remaining studies are distributed equally around days 7–10 post cell inoculation or later time points. The administration schedule was probably the most variable, both from the interval point of view and the final number of administrations (in general, intravenous). It was already shown that the administration protocol may strongly influence outcomes [100,101]. Thus, discrepancy in this factor may help to explain the differences in the results obtained.
4. Immune system. Undesirable interactions between the immune system and nanoparticles can take place, due to either immunostimulation or immunosuppression [102], removing at least part of the administered nanoparticles before their delivery to the target area. Thus, selection of immunocompetent (i.e., mice/rats bearing gliomas originating from their same species) versus immune-deprived (i.e., PDX models or xenograft inoculated with cell lines from human origin) models represents an important step. Moreover, specific pathogen free (SPF) husbandry is a common practice applied in laboratories conducting preclinical experiments, and SPF mice have an immature immune system when compared with wild strains [103]. Therefore, the immune system of SPF mice does not adequately reflect that of clinical subjects, neither do the results [104]. Immunocompetent, syngeneic/isogeneic murine models may be of help, but even in this case, certain cell targets or metabolic pathways may be different from human counterparts, since they will bear murine tumors. The most advisable model would be humanized PDX, although those are more complex and challenging than the already-established murine models [105,106]
5. Animal age. This is a relevant factor that governs baseline immunity and affects the hormone levels (sexual maturity) of the individuals; both impact the disease evolution. However, in most cases indicated in Table 1, the age of individuals is not given, so it was inferred from the standard growth charts. Having this limitation in mind, circa 50% were within 4–6 weeks of age, and the remaining ones were around 7–10 weeks of age, with one study going above (14–18 weeks). These values would rather correspond to early adolescence [107], while glioblastoma incidence increases with age, with circa 50% of the cases diagnosed in patients equal or more than 65 years old [108].
6. Tumor volume. This is an essential parameter, since preclinical tumors are usually variable even when experiments are performed by experienced researchers. This value was reported in circa 60% of the studies included in this review, while it is unclear whether it was not performed or not reported in the remaining studies. Additionally, it is definitely not the same for any therapeutic agent to fight a large established or an early, low-volume, starting tumor [109]. Moreover, based on our experience, we should not assume that the whole cohort will have tumors with comparable volumes.
7. Therapeutic efficacy. Some articles report euthanization of mice and postmortem evaluation of tumors, which may definitely inform about the immediate action of the agent, but ideal situations may imply non-invasive assessment of tumor disappearance or growth arrest at long-term follow-up procedures.

**Table 1:** Nanomedicine-based drug delivery systems tested in orthotopic preclinical glioma models.

Drug(s)	Type of NPs	Animal Model	Age	Immunocompetent/Immunodeprived	Dose and Administration Route	Administration Schedule	Therapy Starting Point	Tumor Volume/Presence Estimated at Starting Point	Targeting	Evaluation of Antitumor Effect/Site Arrival	Refs
TMZ	Liposomes	U87-TL-bearing BALB/c male nude mice	4–6 wk	immuno-deprived	5–10 mg/kg intravenous	Every 3 days, 5 times in total	Day 1 p.i.	yes	Angiopep-2 + anti-CD133 mAb	In vivo bioluminiscence	[110]
TMZ + JQ1	Liposomes	U87-bearing NCR nude mice/GL261-bearing C57/BL6 male mice	6 wk	Both	100 µL intravenous	Every day during 5 days	Day 14 p.i.	yes	Transferrin	In vivo bioluminiscence	[111]
TMZ + ART	Liposomes	TMZ-resistant U251-TR GB nude mice	5–6 wk	immune-competent	5–10 mg/kg intravenous	Every 3 days, 5 times in total	Day 8 p.i.	no	ApoE peptide	In vivo bioluminiscence	[112]
TMZ	Albumin NP	C6-bearing BALB/c and KM mice	5–6 wk	Both	10 mg/kg intravenous	Every 2 days, 8 times in total	Day 5 p.i.	no	Sinapic acid	Histopathology at endpoint	[113]
TMZ	Lactoferrin NP	GL261-bearing C57/BL6 mice	5 to 10 wk *	immune-competent	5 mg/kg intravenous	Every 2 days, 4 times in total	Day 3 p.i.	no	Lactoferrin	Histopathology at endpoint	[114]
TMZ + siTGF	β Polymer-lipid hybrid NP	GL261-bearing C57/BL6 male mice	n.d.	immune-competent	10 mg/kg intravenous	Every 2 days, 3 times in total	Day 8 p.i.	no	Angiopep-2	T2w MRI/ Prussian staining	[115]
TMZ + OTX015	erythrocytemembrane camouflaged nanoparticle	C57BL/6 mice bearing orthotopic GL261-Luc tumor	3–4 wk	Immune-competent	5 mg/kg intravenous	Every 2 days 5 times in total	Day 20 p.i.	no	ApoE peptide	In vivo and ex vivo fluorescence	[116]
Carmustine + O6-Benzylguanine	Polymeric NP	F98-bearing Fischer 344 male rats	7 wk *	immune-competent	6.43–19.29 mg/kg intravenous	Every 4 days, 3 times in total	Day 5 p.i.	no	-----------	T1 and T2w MRI/histopathology at endpoint	[117]
Carmustine + O6-Benzylguanine	Polymeric NP	F98-bearing ICR male mice and F98-bearing male nude mice	5–6 wk *	Both	6.43–19.29 mg/kg intravenous	Every 4 days, 3 times in total	Day 5 p.i.	no	iRGD	Overall survival/In vivo fluorescence	[17]
Carmustine	Magnetic NP	C6-bearing Sprague-Dawley male rats	14–18 wk	immune-competent	0.5–13 mg/kg intravenous, via jugularvein	Single dose	Day 17 p.i.	yes	Magnetic targeting + transient ultrasound-mediated BBB disruption	T1 and T2 * w MRI/Histology	[118]
Carmustine	Micelles	U87-bearing BALB/c male nude mice	5–6 wk *	immune-deprived	1 mg/kg intravenous	Single dose	Day 14 p.i.	yes	T7 peptide	In vivo fluorescence/postmortem brain fluorescence	[119]
Carmustine	Micelles	BT325-bearing BALB/c nude mice	n.d.	immune-deprived	2 mg/kg intravenous	Every 3 days, 5 times in total	Day 14 p.i.	yes	Pep-1 + borneol	In vivo bioluminiscence	[120]
Lomustine	Nanocapsules	U87-bearing female CD-1 nude mice	5–6 wk *	Immuno-deprived	1.2–13 mg/kg intravenous	10 consecutive days	Day 7 p.i.	yes	-------	T2w MRI	[121]
Cisplatin, Oxaliplatin	Liposomes	F98-bearing male Fischer rats	n.d.	Immune-competent	3–5 mg (calculated to body surface area), intracarotid	Single dose	Day 10 p.i.	no	-------	Overall survival/ICP-MS	[122]
Cisplatin	PMAA-PEG Nanogel	101/8-bearing female Wistar rats	9–10 wk *	Immune-competent	5 mg/kg, intravenous (femoral)	Every 5 days, 3 times in total	Day 5 p.i.	yes	mAb anti-Cx43 + mAb anti-BSAT1	T2w MRI	[123]
Cisplatin	PAA-PEG NP	F98-bearing female Fischer344 rats/9L-bearing Sprague-Dawleyrats	8–9 wk *	Immune-competent	2–5 mg/kg, intravenous	Every 7 days, 3 times in total	Day 14 p.i.	yes	- - --	T1w MRI	[124]
Cilengitide	Gelatin-heparin NP	C6-bearing Sprague Dawley male rats	8–10 wk *	Immune-competent	2 mg/kg, intravenous	Every 2–3 days, 8 times in total	Day 7 p.i.	yes	Transient ultrasound-mediated BBB disruption	T1 and T2w MRI	[27]
Cilengitide	Liposomes	C6-bearing Sprague Dawley male rats	8–10 wk *	Immune-competent	2 mg/kg, intravenous	Twice a week, 8 times in total	Day 7 p.i.	yes	Magnetic targeting + transient ultrasound-mediated BBB disruption	T2w MRI and fluorescence imaging/Histology	[125]
Erlotinib + DOX	Liposomes	U87-bearing nude female and male mice	n.d.	Immune-deprived	15.2 µmoles/kg, intravenous	Every 2 days, 3 times in total	Day 10 p.i.	no	Transferrin + Penetratin	Overall survival/Histopathology	[29]
Lapatinib	Albumin NP	U87-bearing BALB/C mice	4–6 wk	Immune-deprived	10–100 mg/kg, intravenous	2–4 times a week, for 2 weeks	Day 8 p.i.	no	- - -	Histopathology	[30]
Nimotuzumab	Methacrylamide NP	U87-EGFRwt-bearing female mice	5 wk	Immune-deprived	5 mg/kg, intravenous	Every other day, 9 times in total	Day 3 p.i.	yes	Choline analogues	In vivo bioluminiscence	[126]
Regorafenib +Disulfiram/cooper	Albumin NP	U87-bearing nude mice GL261-bearing C57/BL6 mice	4–6 wk	Both	1.5 mg/kg, intravenous	Not specified, 5 times total	Day 10 p.i.	yes	Peptide T12 + mannose	In vivo bioluminiscence	[127]
Cediranib +Paclitaxel	PEG-bilirrubin NP	C6-bearing male Balb/c mice	n.d.	Immune-deprived	1.7–3.6 mg/kg, intravenous	Every 2 days, 6 times in total	Day 10 p.i.	no	D-T7 peptide	Histopathology	[128]
Camptothecin	Polymeric NP	U87-bearing athymic nude mice	8 wk	Immune-deprived	4 or 10 mg/kg, intravenous	Every 3 days or every 5 days, 3 times in total	Day 3 or day 5 p.i.	no	Adenosine	Overall survival	[129]
Camptothecin	Polymeric NP	GL261-bearing C57 albino mice	10 wk	Immuno-competent	10–20 mg/kg intravenous,	Every 7 days, 3 times in total	Day 8 p.i.	yes	- - -	In vivo bioluminiscence	[130]
Topotecan	Liposomes	U87, GBM43, or GBM6-bearingFemale athymic mice	6 wk	Immune-deprived	1 mg/kg, intravenous	Twice a week, up to 6 times in total	Day 6–8 pi.i	yes	- - -	In vivo bioluminiscence	[131]
Irinotecan	Liposomes	U251-bearing Rag2 female mice	7–10 wk	“non-leaky” immune-deprived	25–100 mg/kg	Every 7 to 14 days	Day 21 p.i.	no	- - -	Overall survival, histopathology	[132]
Irinotecan + TMZ	Liposomes	U251-bearing NOD.CB17-SCIDfemale mice	7–10 wk	Immuno-deprived	25–50 mg/kg, intravenous	Every 7 days, 3 times in total	Day 14 p.i.	yes	- - -	In vivo fluorescence, overall survival, histopathology	[133]
Irinotecan	Liposomes	U87-bearing male nude rats	7–9 wk *	Immune-deprived	50 mg/kg	Twice a week, 4 times in total	Day 5 p.i.	no	- - -	Overall survival, histopathology	[134]
Irinotecan	Liposomes	GS2-bearing male athymic rats	6 wk	Immune-deprived	3.5 mg, intranasal 0.01 to 1 mg, CED 30 mg/kg, intravenous	Every 7 days, 3 times in total	Unclear15–30 p.i.	yes	- - -	In vivo bioluminiscence	[135]
Irinotecan + Cetuximab	Liposomes	U87-bearing Balb/c nude mice	6–8 wk	Immune-deprived	30 mg/kg, intravenous	Every 3 days, 3 times in total	Day 11 p.i.	yes	Cetuximab + Magnetic targeting	In vivo bioluminiscence	[136]
DOX	Liposomes	U87-bearing male Balb/c nude mice	n.d.	Immune-deprived	2 mg/kg, intravenous	Every 3 days, 5 times in total	Day 6 or day 15 p.i.	no	MC + DA7R	Ex vivo (postmortem) bioluminiscence	[137]
DOX	Liposomes	U87-bearing nude mice	n.d.	Immune-deprived	100 μL with a concentration of 0.01 μM (total dose 10 mg/kg), intravenous	Every 3 days, 5 times in total	Day 10 p.i.	no	CB5005 peptide	Overall survival, Ex vivo (postmortem) bioluminiscence	[138]
DOX + Curcumin	pH-sensitive coreshell NP	C6-bearing male Sprague-DawleyRats	8–10 wk *	Immune-competent	0.33–1 mg/kg, intravenous	Unclear schedule	Day 7 p.i.	yes	----	T1w MRI	[139]
DOX +1-MT i	MSNs	GL261luc-bearing C57BL/6 female mice	6 wk	Immune-competent	2.5 mg/kg	Every 3 days, 5 times in total	Day 5 p.i.	yes	iRGD	In vivo bioluminiscence, MRI	[140]
DOX + HCQ	Legumain responsive gold NP	C6-bearing mice	n.d.	unclear	2.5–15 mg/kg	Every 2 days, 5 times in total	Day 10 p.i.	No	----	Overall survival	[141]

TMZ: temozolomide; CD133: transmembrane glycoprotein overexpressed in cancer stem cells; mAb: monoclonal antibody; JQ1: bromodomain inhibitor; ART: artesunate; iRGD: cyclic integrin-targeting peptide; PMAA: poly (methacrylic acid); OTX01: epigenetic bromodomain inhibitor; PEG: poly (ethylene glycol); PAA: poly (aspartic acid); Cx43: membrane protein connexin 43; BSAT1: brain-specific anion transporter 1; EGFR: endothelial growth factor receptor; MC + D A7R: myristic acid-modified neuropilin-1 and vascular endothelial growth factor-targeting peptide; PDCP-NP: pH-sensitive core-shell nanoparticles; 1-MT: 1-methyltryptophan; DSPE-PEG: 1,2-distearoyl-sn-glycero-3-phosphoethanolamine-N-[methoxy(polyethyleneglycol)-2000; SPION: superparamagnetic iron oxide nanoparticle; HCQ: hydroxychloroquine; MSNs: mesoporous silica nanoparticles; CREKA: fibronectin-targeting peptide. wk: weeks. n.d.: not defined. p.i.: post-inoculation. CED: Convection-enhanced Delivery. * Not detailed, only body weight provided: age estimated according to the body weight charts provided from Jax, Janvier, or Charles River webpages for any specified model.

### 3.1. Representative Examples

All the studies shown in Table 1 prolonged the survival of orthotopic GB rodent models [110,142]. Representative examples are detailed next.

**Figure 5:**
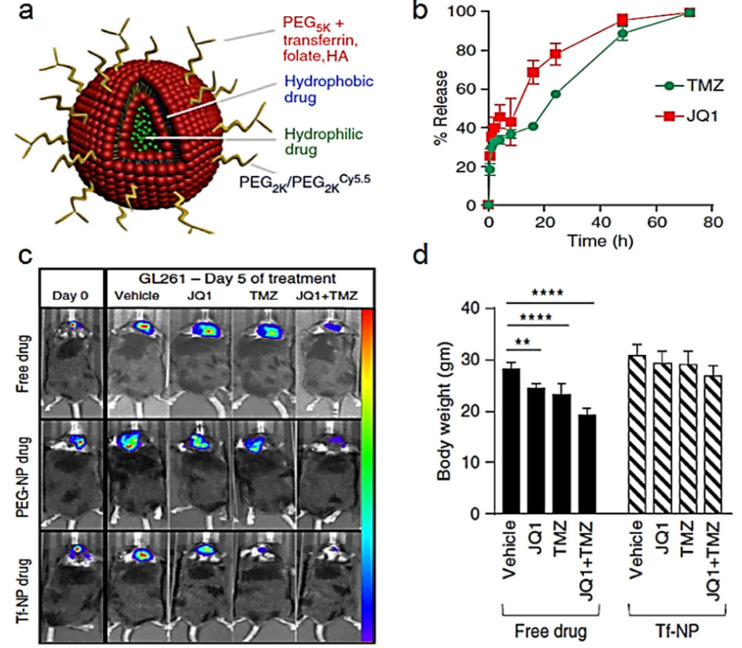
(**a**) Schematic of a PEGylated dual drug (TMZ + JQ1)-loaded liposome that can be functionalized (i.e., transferrin, folate, and hyaluronic acid (HA)) to enhance transport across the BBB and targeting to glioma cells; (**b**) Kinetics of drug release from liposomes loaded with both JQ1 and TMZ; (**c**) Bioluminescent images of GL261 mice taken on day 0 and day 5 following initiation of treatment with free drug formulations (free drug), drugs loaded in PEG-Cy5.5 liposomes (PEG-NP drug), or transferrin-PEG-Cy5.5 liposomes (Tf-NP drug); (**d**) quantification of average daily body weights of mice after 96 h of free drug and Tf-NP treatment course (Student *t*-test for *n* = 5; ** *p* ≤ 0.01; **** *p* ≤ 0.0001). Reproduced from Ref. [111] with permission of the copyright holder.

Temozolomide (TMZ). The efficacy of diverse TMZ-loaded nanocarriers has been evaluated in GB orthotopic animal models. For instance, TMZ-loaded liposomes coated with the BBB-targeting angiopep-2 and the anti-CD133 monoclonal antibody were administered in adult BALB/c nude mice instilled with human glioma stem cells [110]. The median survival time doubled (49.2 days) when compared to the non-structured TMZ (23.3 days), using the same doses (10 mg/kg). Similar nanoparticles based on transferrin-functionalized PEGylated liposomes were also combined with the epigenetic drug JQ1 that blocks the interaction between transcriptional proteins, aiming to sensitize GB towards TMZ. Accordingly, murine glioma-bearing mice treated for 5 days extended median survival while reducing systemic side effects (Figure 5) [111].

Recently, TMZ was encapsulated in ApoE-functionalized liposomes based on artesunate-phosphatidylcholine (ARTPC). This nanosystem was presented as a delivery nanocarrier for dual therapeutic agents (ART/TMZ) to TMZ-resistant U251-TR GB in vivo. In vitro studies showed synergistic DNA damage and induction of apoptosis. Moreover, in vivo studies demonstrated an effective BBB crossing and deep intracranial tumor penetration. The targeted combination liposomes resulted in a significant decrease of U251-TR glioma and improved the survival of mice, reducing systemic TMZ-induced toxicity [112].

Administration of albumin-based NPs coated with a polymer bearing sinapic acid (BBB-delivery shuttle) and encapsulating TMZ to orthotopic C6-bearing BALB/c mice also increased the median survival time while decreasing peripheral toxicity [113]. Encapsulation of TMZ within lactoferrin and administration to orthotopic GL261-bearing C57/BL6 mice also yielded similar results [114]. Lastly, the encapsulation of TMZ in polymer-lipid hybrid nanoparticles decorated with both the targeting peptide angiopep-2 and the small interfering RNA against tumor growth factor β (siTGF-β) demonstrated synergism in modifying the immune microenvironment of GB and improving the effects of the drug [115]. In a recent study, TMZ was coencapsulated with an erythrocyte membrane camouflaged nanoparticle decorated with ApoE peptide [116]. The treatment using the obtained nanoconstructs in mice bearing orthotopic GL261 GB resulted in notable tumor inhibition and extended survival time with few side effects. The efficacy of the treatment was ascribed to several factors, namely, the improved GB targeting due to the greatly enhanced blood circulation time and blood–brain barrier penetration, the synergistic effect in the inhibition of cellular DNA repair and enhanced TMZ sensitivity in tumor cells, and the enhanced antitumor immune responses by inducing immunogenic cell death.

Nitrosoureas. The best therapeutic success of NPs was reached by combining nitrosoureas with other drugs [17,117] in comparison to monotherapy [118,119,120]. On top of that, higher doses can be administered as encapsulation, and solubility is increased in comparison to free drugs, prolonging in this way the survival of tumor-bearing mice decreasing/abrogating myelosuppressive effects [121].

Platinum-based drugs. Lipoplatin and Lipoxal, which are liposomal formulations containing cisplatin and oxaliplatin, respectively, were administered to F98 glioma-bearing male Fischer rats and demonstrated boosted anticancer activities compared to free drugs [122]. PEGylated poly (methacrylic acid) nanogels loaded with cisplatin and superficial monoclonal antibodies were also i.v. administered to glioma-bearing female Wistar rats, and the results indicated that nanogels exhibited enhanced tumor growth inhibition and increased the life expectancy of animals in comparison with other investigated formulations [123]. Moreover, an enhanced delivery efficiency and survival of animals, while reducing off-target toxicity, was obtained by using MRI-guided ultrasound after i.v. administration in glioma-bearing rats facilitating BBB crossing [124].

EGFR inhibitors. Administration of cilengitide-loaded NPs in C6-bearing Sprague–Dawley rats with BBB disrupted by transient focused-ultrasound [27], or directed with magnetic targeting [125] attained a median survival of up to three times that detected for free cilengitide, with no pathological damage to major organs. In another example, the authors demonstrated that the coencapsulation of erlotinib with DOX in liposomes functionalized with transferrin and cell-penetrating peptides was able to enhance its therapeutic effect [29]. Other approaches involving the encapsulation—within NPs—of tyrosine kinase inhibitors, such as nimotuzumab, cediranib, or regorafenib [126,127,128], endorsed BBB crossing and glioma cellular uptake, therefore increasing the therapeutic effectiveness.

Topoisomerase inhibitors. Polymeric NPs and liposomes decreased secondary effects of topoisomerase inhibitors, such as camptothecin and its derivatives topotecan and irinotecan [129,130,131,132,133,134,135,136], while providing a significant median survival increase [136]. Nanoliposomal formulations encapsulating irinotecan were particularly interesting when tested in preclinical studies with male athymic rat models [135] using three different administration routes, i.e., i.v., CED, and intranasal administration (IN). All of them were tolerated by non-tumor-bearing animals even though CED and IN allowed for a 4-fold and 100-fold dose range increase, respectively. Afterwards, the experiments were repeated on tumor-bearing animals showing significant survival benefits, though this strongly depended on parameters such as infusate volume, addition rate, and number of infusions. The conclusion suggests that a single treatment of CED had the same effect on overall survival than multiple IV treatments. Interestingly, IN administration showed significantly increased survival of animals and demonstrated the promising potential of this non-invasive approach of delivery bypassing the BBB using nanoliposomal formulations for the treatment of brainstem gliomas. These excellent results led to human clinical trials (NCT03086616, finished last October 4th), with no results reported yet.

Finally, DOX-loaded PEGylated liposomes covered with myristic acid modified DA7R were i.v. administered to intracranial U87-bearing male nude mice showing therapeutic effect [137]. If the DOX-loaded PEGylated liposomes are decorated with the CB5005 peptide, similar results are obtained with remarkable cell and nucleus permeabilities, resulting in a significant increase of the survival times [138]. Finally, different examples of NPs combining DOX with a curcumin-loaded dual-layer pH-sensitive NPs [139], 1-methyltryptophan (immune checkpoint inhibitor) in mesoporous silica [140], or hydroxychloroquine-loaded legumain-responsive gold nanoparticles [141] have been reported and substantiated their efficiency to overcome BBB, while enhancing synergistic effects.

Other interesting explorative studies can be found in literature. For example, an innovative development consisted in biomimetic designs including angiopeptide-2 functionalized red blood cell membrane (Ang-M) camouflaging of NPs carrier containing copper chelators (Dp44mT), administered to Cu-enriched orthotopic U87MG-Luc GB for glioblastoma treatment. The principal objective of this study was to induce the generation of tumor-toxic reactive oxygen species (ROS) by selective targeting of copper present in cancer cells. The experimental results indicated that the NPs actively targeted and traversed the BBB, delivering Dp44mT specifically to GB cells and substantially prevented orthotopic GB growth, leading to maximal increases in median survival time [143]. Another interesting development was based in the encapsulation of Plk1 inhibitor volasertib (Vol) in an angiopep-2 functionalized polymersome prepared from poly(ethylene glycol)-b-poly(L-tyrosine)-b-poly(L-aspartic acid). The in vitro assays determined an eight-fold better antitumor activity, and in vivo studies demonstrated an effective suppression of tumor growth in orthotopic U87 MG GB mice, increasing survival rates and reducing toxicity in comparison with free Vol [144].

It is worth to mention that nanomedicines are attractive systems, but the currently developed nanomedicines often suffer from poor serum stability or premature drug release. In this sense, the development of liposomes or polymeric nanoparticles based on crosslinking strategies to employ stimuli-responsive chemical bonds were performed [145]. These improvements allow holding a robust nanostructure during systemic circulation, releasing the payloads spatiotemporally in a controlled manner in response to particular tumoral conditions.

Apart from the later representative examples, there are several other nanosystems based in inorganic nanoparticles with potential to be used in GB treatment. Metallic nanoparticles are the most used approach for GB among inorganic nanoparticles, since they have shown high internalization ability, with the potential to be used as radiotherapy enhancers and as agents for brain imaging, presenting a good strategy as theragnostic probes. Some examples are related to metal oxide [146,147], gold [148], and silver [149] nanoparticles. Despite efforts made with metallic nanoparticles, few studies have validated BBB crossing for in vivo GB treatment [150], suggesting that more studies are necessary with this type of nanoparticles. Mesoporous silica nanoparticles (MSNs) are also inorganic NP with emerging uses in GB treatment due to their drug loading capacity, low toxicity, excellent biodistribution, and ease for surface functionalization, leading to tumor targeting, among others [151]. Several studies have shown that MSNs might have GB therapeutic potential. However, the lack of in vivo studies limits the projection of these type of nanoparticles [152,153]. Interestingly, carbon allotropes, such as carbon nanotubes (CNTs), graphene-based nanomaterials (GBNs), and quantum dots (QDs), have also been tested for GB treatment. CNTs garnered superior properties such as hydrophilicity, biocompatibility, and specificity, and a great variety of chemotherapeutics were encapsulated for GB treatment [154,155,156]. GBN, graphene oxides (GO), and reduced graphene oxides (rGO) can be functionalized with specific molecules on their surface to enhance BBB crossing [157,158], as well as different drugs with activity against GB [159], indicating that functionalization could significantly improve the BBB transport and GB targeting specificity. Finally, QDs have demonstrated to be nanoconstructs able to be functionalized with different targeting molecules and drugs, and showed remarkable cytotoxicity against GB cells, displaying synergistic effects owing to the dual-drug combinations [160,161,162]. However, as in most studies with inorganic nanoparticles, the lack of in vivo studies makes it difficult to evaluate the real potential of these nanosystems for the treatment of GB. 

### 3.2. Targeting and Theranostic

Beyond the examples summarized in Table 1, another area of activity is the decoration of nanoparticles with moieties binding BBB target proteins. Since passive targeting drug delivery systems have not shown clinical value due to a lack of selectivity, many developments are focused on active targeting drug delivery nanosystems to achieve selective treatment of GB. These nanoconstructs are designed to exploit differences in receptors or antigen expression on the surface of tumor cells in comparison to normal cells. Common active targeting vectors include small molecules, proteins, peptides, and aptamers, such as folic acid, transferrin, and the RGD peptide [163]. 

**Figure 6:**
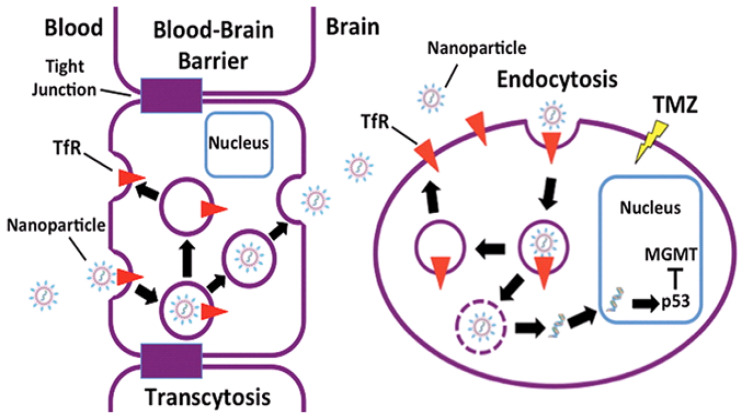
Schematic of BBB crossing ability of drug-loaded cationic liposomes and cancer cell internalization induced by surface functionalization with antitransferrin receptor (TfR) antibody fragment designed to target TfR. Reproduced from Ref. [167] with permission of the copyright holder.

However, special care must be considered, since most of the time such proteins are not exclusively expressed at the brain microvasculature. Therefore, finding alternative strategies to target the BBB are required to increase drug efficacy [164]. In fact, targeting of liposomes towards the glioma peritumoral area dates to 2012 [165], with several other examples being reported since then. For instance, Kim et al. reported siRNA and small drug-loaded cationic liposomes functionalized with an antibody recognizing the transferrin receptor, resulting in improved effect when compared to free TMZ (see Figure 6) [166,167]. The treatment of U87 or TG98 xenografts with TMZ-containing NPs decorated with transactivator of transcription (TAT) peptides and/or imaging contrast agents exhibited enhanced effect on features such as tumor volume, Ki67 staining, apoptosis, and CD133 staining, as well as increased survival [168].

Further experimental designs involved emulsions encapsulating CPT, tetraethylene glycol (TEG), and α-lipoic acid (ALA), which released the active drug upon enzymatic degradation [169]. In vitro assays using U87 MG glioma cells revealed a notable intracellular uptake via cell membrane penetration, whereas in vivo studies demonstrated that the NPs were able to cross the BBB and to increase survival rates in a U87 xenograft model. Alternatively, endogenous exosomes are more stable than liposomes, and thus, their use may improve forthcoming drug delivery approaches. Beyond targeting, the development of theranostic NPs, such as iron oxide NPs (IONPs) that contribute not only to therapy, but are also able to act as MRI imaging agents, has been explored. For example, IONPs coated with chitosan, PEG, and polyetheleneimine (PEI) exhibited T_2_ MRI contrast and siRNA delivery that fruitfully knocked down Ape1 expression, an enzyme that is crucial in the base excision repair pathway [170]. 

Alternatively, Mu et al. described IONPs decorated with gemcitabine (GEM), hyaluronic acid, and chlorotoxin (CTX), enabling GB cell targeting [171], even though hindering its infiltrative nature, which currently represents a challenge for thorough surgical resection [172,173]. PEG-coated IONPs have also been functionalized with paclitaxel (PTX), fluorescein (FL), and CTX to eliminate in vitro MGMT-resistant GB tumor cells [174]. 

Despite the relevant scientific advances, the protective effect of the BBB poses challenges to the clinical treatment of gliomas, even when its integrity is disrupted as in GB. Within the most recent progresses, the development of biomimetic drugs/materials with homologous binding and immune escape functions has been regarded as the most promising alternative. Accordingly, future research should pay attention to the latest progress in basic research, including structural characteristics, composition, and permeability of BBB in gliomas. Additionally, researchers should identify and evaluate the factors that may affect the in vivo behavior of a specific nanosystem, such as the biodegradability of the utilized nanoparticles and aspects related to the protein corona on the nanoparticle surface, which can vary depending on the tumor type, tumor environment conditions, and even the administration route chosen for treatment. Although many nanosystems are designed in order to minimize this impact, it must be considered, and the take-home message may be that in vitro experiments are not able to totally mimic in vivo conditions affecting the protein corona.

## 4. Clinical Studies

The average rate of translation from preclinical models to clinical cancer trials is less than 8% [175], mostly because the lack of: (i) standardization protocols, (ii) experimental details regarding cohorts, and (iii) best practice standards. A summary of them is given in Table 2.

Among them, worth to mention are the aminosilane-coated superparamagnetic iron oxide NPs, termed NanoTherm^®^, which generate heat upon application of an external alternating magnetic field that induces thermal ablation of the tumor tissue [178]. Accordingly, in a non-randomized phase 2 trial, the use of these NPs doubled the median overall survival of patients (up to 13.4 months) [65,179] with recurrent GB. Thus, NanoTherm^®^ was approved in 2010 by the European Medicines Agency (EMA), though patients were re-irradiated as an adjunctive therapy to nanoparticles. Another example of inorganic nanocarriers suitable to reach clinical trials (NCT03020017, phase I, active) are gold NPs functionalized with nucleic acids targeting the gene BCL2L12 associated with GB tumor growth [180].

Beyond these two inorganic nanocarriers, most clinical trials are based on liposomal systems. FDA-approved Onyvide^®^ consists of a PEGylated irinotecan liposomal formulation, aimed to treat metastatic pancreatic cancer, and already investigated in four clinical trials (NCT03119064, NCT00734682, NCT02022644, and NCT03086616), either alone or combined with TMZ and administered via i.v. or CED. Another PEGylated liposomal doxorubicin formulation (Caelyx^®^, Doxil^®^) has been tested following the Stupp regimen [6] in the RNOP-09 phase II clinical trial (NCT00944801); however, this results in a non-reliable median OS, most likely owing to the insufficient sample size (*n* = 63) [181,182]. DOX-bearing PEGylated liposomal formulations combining additional glutathione (NCT01386580; phase I/II clinical trial) or cetuximab (NCT03603379, phase I, recruiting) were also analyzed. Other liposomes were focused on gene-based therapies, such as the plasmid-loaded cationic liposome bound to anti-TfR antibodies (SGT-53), which, combined with TMZ, were able to finalize a phase II clinical trial (NCT02340156) for recurrent GB treatment. The same system lately joined a phase I trial (NCT03554707) to treat CNS tumors in children. Moreover, a related formulation (SGT94-01), based on the RB94 gene encapsulated in a liposome and bearing antitransferrin receptor single chain antibody fragment targeted to tumor cells, was tested on solid tumors (NCT01517464).

## 5. Summary and Future Perspectives

Up to now, satisfactory results increasing median survival have been obtained using NPs for the treatment of GB in preclinical models, endorsed by the potential of NPs to increase drug availability with both lower doses and side effects. Functionalization of NPs with targeted receptors and/or proteins overexpressed on GB cells have also demonstrated in vivo capabilities regarding the specific delivery of drugs to tumor cells. However, only a few of them (less than 8%) have reached Phase I/II clinical trials and almost none of them have progressed to phase III or even to randomized phase II trials, which is surprising considering the successful preliminary preclinical results. This could be at least partially attributed to aspects related to the lack of standardization in preclinical studies. In other words, the use of NPs still must face development and planification challenges before its complete clinical implementation becomes true. Other issues to be solved are effective BBB crossing, minimizing side effects, and avoiding inappropriate biodistributions. Additionally, human biological diversity and disease heterogeneity should also be considered, as well as age and gender aspects, at least in preclinical models [183].

Future strategies should also reconsider the physicochemical and colloidal properties of the NPs, which are currently still not fully agreed. In other words, it would be highly recommended to carry out detailed and systematic preclinical studies that helps us to unequivocally determine and universally agree on basic aspects, such as dose ranges, administration schedules, pharmacokinetics and absorption, biodistribution, metabolism, and excretion, before reaching any clinical trial. Nanoformulations should also prove sufficient long shelf life and stability during long-term storage and clinical administration. 

On top of that, alternative and more effective administration pathways should be fully studied to avoid physiological barriers (i.e., BBB). In this context, intranasal drug administration represents a non-invasive route to directly targeting the brain. In the last few years, a few nanomedicines have been developed for this type of nose-to-brain transport that bypasses the BBB and reduces systemic side effects. The preclinical and clinical studies using this administration route predict a breakthrough advancement in the fight against GB and have demonstrated the benefits of intranasal administration versus classical routes. In fact, our research groups have initiated this type of preclinical studies with very promising results, which encourage the developing of advanced and improved nanomedicines for GB treatment [184,185].

## 6. Conclusions

Current treatment strategies for glioblastoma are clearly unsuccessful, resulting in low patient survival rates and poor prognosis. Nanomedicine is able to afford different tools for improving diagnosis and especially to enable efficient targeting and transport of drugs across the BBB. Although many nanoformulations are currently being evaluated in cell lines and animal models, only a few have reached the clinical trial stage, and none of them proved to be an effective replacement for the present standard of care. The possible reasons behind this deceiving outcome are the hurdles due to the BBB barrier and the immunosuppressive glioblastoma environment as well as its heterogeneity. However, recent results are providing hope about the nanomedicine potential for achieving a better percentage of long-term survivors, provided some relevant experimental aspects are taken into account.

Present advances in glioblastoma therapy may help to understand the molecular mechanisms underlying tumor progression and the potential role of new nanomedicine approaches. This review has discussed the advantages and limitations of current developments and aimed to pave the way for research in the forthcoming years. There is presently no consensus about the most effective nanoformulations for brain cancer treatment, and variability in nanoparticle size, composition, targeting ability or mechanisms of drug delivery. This situation makes it extremely hard to compare different studies. In this scenario, an effort to standardize in vitro/in vivo studies is urgently needed. Moreover, the ongoing clinical trials and future studies will hopefully shed some light on the optimal nanoparticle design for improving outcome in brain tumor patients. Thus, the major challenge of next years will be to optimize the existing nanoformulations and/or to work towards additional improvements, including the investigation of new administration routes, to enhance the translatability of therapies.

## Figures and Tables

**Table 2:** Nanoparticle-based systems containing drugs currently undergoing clinical trials for glioma treatment. TMZ: temozolomide; anti-TfR: antitransferrin.

Name	Drug	Particle Type	Targeting Moieties	Clinical Trials.Gov Identifier
Onyvide^®^	Irinotecan	PEGylated liposomes	- - -	NCT03119064
NL CPT-11	Irinotecan	PEGylated liposomes	- - -	NCT00734682
Caelix^®^	DOX (combined with prolonged TMZ)	PEGylated liposomes	- - -	NCT00944801
2B3-101	DOX	PEGylated liposomes	Glutathione	NCT01386580
C225-ILs-Dox	DOX	Liposomes	Cetuximab	NCT03603379
Nanotherm^®^	- - -	Iron oxide nanoparticles	- - -	Magforce, Inc. (Berlin, Germany) (Approv. 2013)
SGT-53	P53 plasmid (combined with oral TMZ)	cationic liposomes	anti-TfR antibody	NCT02340156 NCT03554707
SGT94-01	RB94 plasmid	Liposomes	anti-TfR antibody	NCT01517464
(NU-0129)	- - -	gold nanoparticles	nucleic acids targeting BCL2L12 gene	NCT03020017
DaunoXome^®^	- - -	Liposomes	- - -	(Zucchetti et al.) [176]
Myocet^®^	- - -	Liposomes	- - -	NCT02861222 (Chastagner et al.) [177]
